# Low transverse incision for lateral neck dissection in patients with papillary thyroid cancer: improved cosmesis

**DOI:** 10.1186/s12957-017-1160-1

**Published:** 2017-05-04

**Authors:** Chang Myeon Song, Yong Bae Ji, In Sik Kim, Ji Young Lee, Dong Sun Kim, Kyung Tae

**Affiliations:** 1 Department of Otolaryngology-Head and Neck Surgery, College of Medicine, Hanyang University, 222 Wangsimni-ro, Seongdong-gu, Seoul 04763 South Korea; 2 Department of Radiology, College of Medicine, Hanyang University, 222 Wangsimniro, Seongdong-Gu, Seoul 04763 South Korea; 3 Department of Internal Medicine, College of Medicine, Hanyang University, 222 Wangsimniro, Seongdong-Gu, Seoul 04763 South Korea

**Keywords:** Papillary thyroid carcinoma, Incision, Lateral neck dissection, Selective neck dissection, Lymph node dissection

## Abstract

**Background:**

Various incisions and approaches have been developed for lateral neck dissection. The purpose of this study was to compare the surgical and cosmetic outcomes of a single low transverse incision with the hockey stick incision for lateral neck dissection (LND) in patients with papillary thyroid carcinoma (PTC).

**Methods:**

We retrospectively analyzed 97 patients with PTC who underwent therapeutic LND and total thyroidectomy by low transverse incision (62 patients) or hockey stick incision (35 patients). We compared the operative results, cosmetic outcomes, objective scar measurement, and sensory disturbance between the two groups.

**Results:**

The number of harvested and metastatic lymph nodes, Vancouver Scar Scale scores, and sensory change were not significantly different between the two groups. The mean number of harvested lymph nodes in level II was 9.82 vs. 9.63 (*P* = 0.885) (transverse incision vs. hockey stick incision, respectively) and in level V was 6.36 vs. 5.63 (*P* = 0.597). However, subjective satisfaction with the scar and neck contour was higher in the low transverse incision group compared with the hockey stick incision group. Scores for scar consciousness and sensory change were not significantly different between the two groups.

**Conclusions:**

A single low transverse incision may provide equivalent surgical outcomes and superior cosmetic outcomes compared with the hockey stick incision for LND in PTC.

## Background

The incidence of papillary thyroid carcinoma (PTC) is increasing due to advances in screening modalities, including ultrasonography [1]. Although PTC has a good prognosis, cervical lymph node metastasis is frequently detected, which increases locoregional recurrence and decreases survival, particularly when the metastasis is in the lateral compartment lymph node [2, 3]. Therapeutic lateral neck dissection (LND) should be performed in patients with PTC when lymph node metastasis is in the lateral compartment [4]. The compartment-oriented en bloc neck dissection is favored over a “berry-picking procedure” for lateral compartment node metastasis. The extent of LND includes modified radical neck dissection (MRND), selective neck dissection including levels II, III, IV, and V, or super-selective neck dissection [5].

Several types of incision can be used for LND of patients with PTC, including a hockey stick incision, an apron incision, a single transverse incision, a modified MacFee incision, or a modified Schobinger incision [6, 7]. In practice, the most commonly used incisions are the hockey stick incision, the modified MacFee incision, and the low transverse incision. A hockey stick incision provides excellent access to neck levels I and II, but invades the relaxed skin-tension line and can result in scar hypertrophy [8]. A single low transverse incision is parallel to, and does not invade, the relaxed skin-tension line, and therefore usually favors a good postoperative scar. However, access to the upper neck levels, including levels I and II might be more difficult with low transverse incision than with other incisions due to the increased distance from the incision site. These days most surgeons prefer to use a transverse incision, with or without modifications, for LND in patients with PTC due to the potential advantage of superior cosmesis. However, the efficacy of a low single transverse incision for LND has not yet been evaluated in comparison with other incision approaches.

In the current study, we compared surgical outcomes, including objective scar assessment, subjective cosmetic satisfaction, sensory change, lymph node yield, and surgical completeness, between low transverse incisions and hockey stick incisions following LND for patients with PTC and lateral compartment node metastasis.

## Methods

We analyzed the data of 97 patients with PTC who underwent unilateral therapeutic selective neck dissection with total thyroidectomy and central neck dissection in a tertiary hospital by a single surgeon (K.T.) between January 2010 and September 2014. Our eligibility criteria included patients with PTC who had undergone unilateral therapeutic lateral compartment neck dissection with concomitant total thyroidectomy, whose pathologic results confirmed lateral compartment lymph node metastasis, and who had completed both a scar assessment and a questionnaire to evaluate cosmetic satisfaction and sensory change. We excluded patients who had undergone bilateral LND and those with other pathologic types of thyroid cancer, distant metastasis, recurrent tumor, or a history of previous neck irradiation or neck surgery.

Of the 97 patients, 62 patients underwent LND by single low transverse incision, and 35 patients by L-shaped hockey stick incision. No randomization was performed between the two groups. The choice of skin incision was mainly decided according to surgeon’s preference and patient status.

**Fig. 1:**
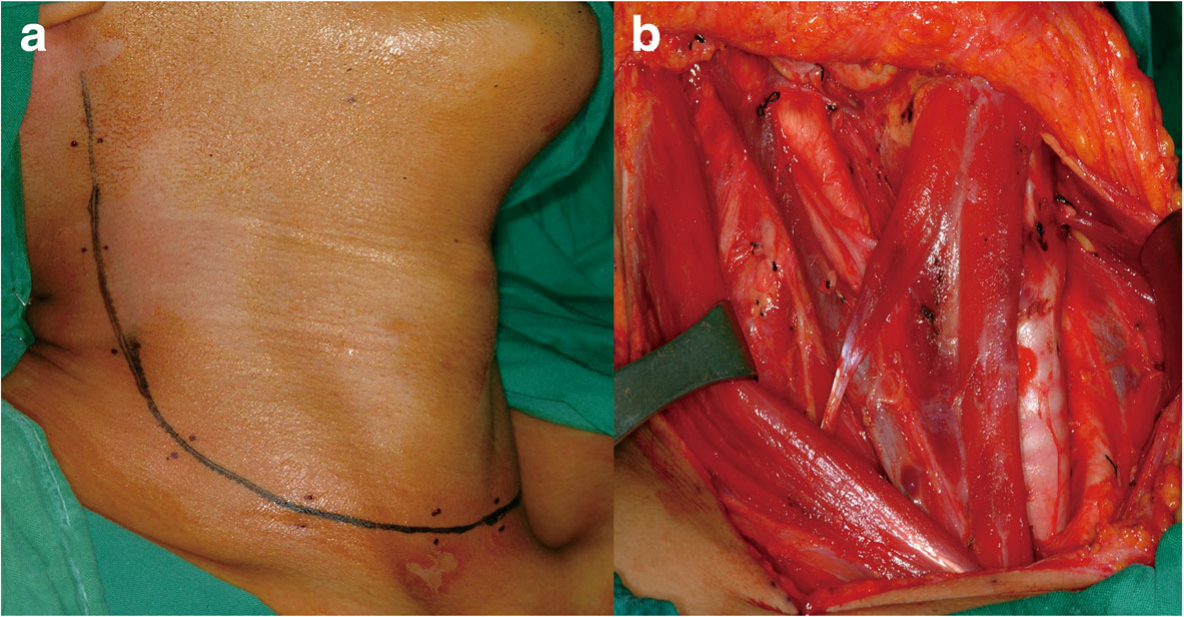
Hockey stick incision for right lateral neck dissection. **a** The incision is composed of a low transverse incision and a vertical extension superiorly reaching the mastoid tip. **b** The surgical view of the entire neck, including upper neck levels I and II

**Fig. 2:**
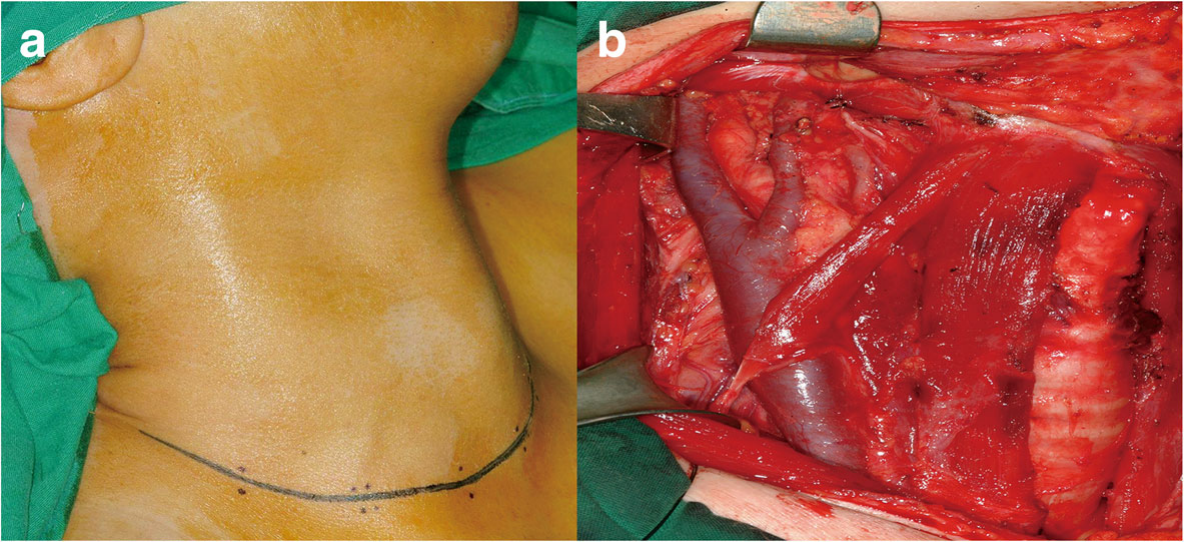
Single low transverse incision for right lateral neck dissection. **a** The incision is located one finger-breadth above the suprasternal notch and parallel to the skin-tension line, from the medial border of the contralateral sternocleidomastoid muscle to the anterior border of the ipsilateral trapezius muscle. **b** The surgical view after total thyroidectomy and lateral neck dissection: complete dissection of levels II to V is possible

In our institution, a hockey stick incision is a transverse incision one finger-breadth above the suprasternal notch with an extension superiorly lateral to the posterior border of the sternocleidomastoid muscle, reaching the mastoid tip (Fig. 1a, b). A single low transverse incision is placed one finger-breadth above the suprasternal notch and extends parallel to the skin-tension line from the medial border of the contralateral sternocleidomastoid muscle to the anterior border of the ipsilateral trapezius muscle (Fig. 2a, b).

In our department, neck levels II to V were routinely dissected in most patients undergoing LND. Level I was not dissected unless there was suspicious lymph node metastasis. Neck levels IIb and Va were also not dissected in some patients with either no suspicious lymph nodes in level IIa or single-level lymph node metastasis in preoperative imaging or intraoperative finding. We did not perform prophylactic LND for patients with PTC who were clinically negative in the lateral compartment. The lateral compartment lymph node specimens removed were classified according to nodal level, including the sublevels IIa, IIb, Va, and Vb. In all cases, the platysma layers and subcutaneous tissue layers of the upper and lower skin flap were sutured with an absorbable polyglactic acid suture material (4.0 Vicryl^TM^; Ethicon, Inc.), and skin closure was performed with a non-absorbable monofilament Nylon suture (5.0 Ethilon^TM^, Ethicon, Inc.). All non-absorbable sutures were removed 6 days after the operation.

We compared the demographics, tumor characteristics, surgical outcomes (including operative time, amount of drainage, perioperative complications, number of harvested, and metastatic lymph nodes according to neck levels), level of thyroid-stimulating hormone (TSH)-stimulated serum thyroglobulin (Tg), and iodine uptake in a whole-body iodine scan between the two groups. Postoperative radioactive iodine (RAI) ablation was performed 2–4 months after surgery using a dose of 30–150 mCi. A diagnostic RAI whole-body scan was performed 6–12 months after the first RAI ablation with a dose of 2–30 mCi both with and without a therapeutic purpose. TSH-stimulated serum Tg level was measured during RAI ablation and during a diagnostic whole-body iodine scan.

A scar was analyzed objectively using the Vancouver Scar Scale (VSS) at 12–24 months postoperatively. VSS is a validated objective scar scale that is commonly used in clinical studies [9]. VSS assesses four domains: vascularity, pigmentation, pliability, and height of the scar [10]. Vascularity, pigmentation, and height of the scar are scored from 0 to 3, and pliability is scored from 0 to 5. The score is the sum of four domains, with a score of 0 representing normal skin and a score of 14 representing the worst scar [11].

Subjective cosmetic satisfaction and consciousness of scar were evaluated at the same time as VSS using an author-developed questionnaire. Cosmetic satisfaction was assessed with two questions: (1) How satisfied are you with the neck scar? (2) How satisfied are you with the contour of the neck? Each question was scored as 0 (very satisfied), 1 (satisfied), 2 (average), 3 (dissatisfied), and 4 (very dissatisfied) [11]. Scar consciousness was assessed with four questions: (1) Do you think others look at your neck scar? (2) Do you try to conceal your scar? (3) Does your neck scar influence your choice of clothes? (4) How often do you think of your scar? Each question was scored as 0 (never), 1 (sometimes), 2 (frequently), or 3 (always) [11]. Total cosmetic satisfaction score was defined as the sum of the scores for cosmetic satisfaction (two questions) plus the sum of the scores for scar consciousness (four questions).

Sensory change was evaluated at the same time using a questionnaire in which patients were asked if they have any hypesthesia or paresthesia in the neck: Do you have decreased sensation or paresthesia in your neck area [12]? Score of sensory change ranged from 0 (none) to 4 (severe). All VSS assessments and the questionnaires for cosmesis and sensory disturbance were conducted by the same head-and-neck oncology nurse coordinator.

Differences in continuous variables were analyzed with the Student’s *t* test. Differences in categorical variables were analyzed with the chi-squared test or Fisher’s exact test when the cell size was small. All statistical analyses were performed using SPSS version 21.0 (SPSS Chicago, IL). A *P* value <0.05 was considered statistically significant.

## Results

Demographics and tumor characteristics are listed in Table 1. Age and gender of the patients were not significantly different between patients in the transverse incision group and those in the hockey stick incision group. Primary tumor size, rates of multiple and bilateral tumor, minimal extrathyroidal extension, lymphovascular invasion, tumor (T) and node (N) classification, and tumor, node, metastasis (TNM) staging were not significantly different between the two groups.

**Table 1:** Patient and tumor characteristics in patients with low transverse lateral incision and hockey stick incision

Parameter	Transverse Incision group *n* = 62	Hockey stick Incision group *n* = 35	*P* value
Age	47.2 ± 13.3	46.7 ± 14.6	.866
Gender			
Female	41/62 (66.1%)	22/35 (62.9%)	.826
Size of tumor (mm)	19.3 ± 13.5	24.1 ± 16.5	.129
Multiplicity of tumor	33/62 (53.2%)	12/35 (34.3%)	.091
Bilaterality of tumor	20/62 (32.3%)	11/35 (31.4%)	.560
Minimal ETE	30/62 (48.4%)	19/35 (54.3%)	.674
Lymphovascular invasion	28/62 (45.2%)	22/35 (62.9%)	.138
T classification			.194
T1	18 (29.0%)	5 (14.3%)	
T2	2 (3.2%)	4 (11.4%)	
T3	41 (66.1%)	24 (68.6%)	
T4	1 (1.6%)	2 (5.7%)	
N classification			NA
N1b	62 (100%)	35 (100%)	
TNM stage			.503
I	29 (47.5%)	17 (48.6%)	
II	0 (0%)	0 (0%)	
III	31 (50.8%)	16 (45.7%)	
IV	1 (1.6%)	2 (5.7%)	

*ETE* extrathyroidal extension, *NA* not applicable

Comparisons of the surgical outcomes between the two groups are summarized in Table 2. The extent of central and lateral neck dissection, operative time, and amount of drainage were not significantly different between the two groups. The rates of complications including hypoparathyroidism, vocal cord paralysis, hematoma, seroma, chyle leakage, and wound infection were not significantly different. There was no injury to the spinal accessory nerve, brachial plexus, or phrenic nerve in either group. The incidence of postoperative RAI ablation and the dose of RAI were not significantly different. TSH-stimulated serum Tg levels at the first RAI and at diagnostic whole-body iodine scan, and uptake outside the thyroid bed on whole-body iodine scan were not significantly different between the two groups.

**Table 2:** Comparison of surgical outcomes of patients with low transverse lateral incision and hockey stick incision

Parameter	Transverse Incision group *n* = 62	Hockey stick Incision group *n* = 35	*P* value
Total thyroidectomy	62 (100%)	35 (100%)	NA
Central neck dissection			.604
Unilateral	5 (8.1%)	3 (8.6%)	
Bilateral	57 (91.9%)	32 (91.4%)	
Lateral selective neck dissection			.132
Level II, III, IV, V	44 (71.0%)	29 (82.9%)	
Level II, III, IV	11 (17.7%)	5 (14.6%)	
Level III, IV, V	7 (11.3%)	1 (2.9%)	
Total operative time (min)	273 ± 79	270 ± 90	.849
SND (II–V)	276 ± 94	290 ± 83	.504
SND (II–IV)/SND (III–V)	230 ± 48	239 ± 65	.746
Amount of drainage (mL)	386 ± 215	410 ± 233	.632
Complication			
Hypoparathyroidism			.916
Transient	24/62 (38.7%)	13/35 (37.1%)	
Permanent	1/62 (1.6%)	1/35 (2.9%)	
Vocal cord paralysis			.619
Transient	6/62 (9.7%)	4/35 (11.4%)	
Permanent	1/62 (1.6%)	0/35 (0%)	
Hematoma	3/62 (4.8%)	2/35 (5.7%)	.597
Seroma	2/62 (3.2%)	2/35 (5.7%)	.618
Chyle leakage	2/62 (3.2%)	0/35 (0%)	.534
Spinal accessory nerve injury	0	0	NA
Horner syndrome	0	0	NA
Brachial plexus injury	0	0	NA
Phrenic nerve injury	0	0	NA
Wound infection	0	2/35 (3.2%)	.534
Skin necrosis	0	0	NA
Follow-up period (month)	60.6 ± 26.3	58.0 ± 24.9	.640
RAI ablation	60/62 (96.8%)	31/35 (88.6%)	.184
Dose of RAI ablation (mCi)	153.4 ± 32.7	161.1 ± 34.1	.344
Stimulated Tg at the first RAI (ng/ml)	7.35 ± 12.0	6.78 ± 9.70	.820
Stimulated Tg at diagnostic WBS after RAI (ng/ml)	1.02 ± 1.62	1.15 ± 2.25	.741
Uptake outside thyroid bed at WBS after RAI	0/60	1/31 (3.2%)	.340

Values in the table are mean ± SD, unless indicated otherwise

*SND* selective neck dissection, *NA* not applicable, *RAI* radioactive iodine, *Tg* thyroglobulin, *WBS* ^131^I whole-body scan, *SD* standard deviation

The numbers of harvested and metastatic lymph nodes are listed in Table 3. The number of harvested and metastatic lymph nodes was not significantly different in total, in the lateral compartment, or in each neck level including levels II, III, IV, V, and VI. The mean number of harvested lymph nodes in level II was 9.82 vs. 9.63 (*P* = 0.885) (transverse incision vs. hockey stick incision, respectively) and in level V was 6.36 vs. 5.63 (*P* = 0.597).

**Table 3:** Number of harvested and metastatic lymph nodes in patients who received lateral neck dissection

Parameter		Transverse Incision group *n* = 62	Hockey stick Incision group *n* = 35	*P* value
Total	Harvested LN, *n*	36.67 ± 19.99	32.06 ± 16.34	.263
	Metastatic LN, *n*	10.29 ± 7.61	8.87 ± 6.73	.378
	Ratio (%)	30.74 ± 17.70	28.30 ± 20.55	.550
Central (level VI)	Harvested LN, *n*	10.07 ± 8.45	7.83 ± 6.79	.214
	Metastatic LN, *n*	5.05 ± 4.71	3.60 ± 2.84	.126
	Ratio (%)	52.94 ± 31.62	61.08 ± 34.57	.271
Lateral	Harvested LN, *n*	27.70 ± 15.49	29.29 ± 10.97	.631
	Metastatic LN, *n*	5.65 ± 4.09	6.52 ± 4.89	.393
	Ratio (%)	23.98 ± 17.33	22.87 ± 15.90	.777
Level II	Harvested LN, *n*	9.82 ± 6.12	9.63 ± 4.49	.885
	Metastatic LN, *n*	2.42 ± 1.82	2.33 ± 2.05	.848
	Ratio (%)	33.63 ± 32.72	24.83 ± 22.99	.243
Level III	Harvested LN, *n*	7.96 ± 4.72	8.48 ± 3.20	.609
	Metastatic LN, *n*	2.06 ± 1.89	2.11 ± 2.04	.906
	Ratio (%)	28.49 ± 27.72	24.69 ± 23.86	.545
Level IV	Harvested LN, *n*	8.13 ± 4.96	8.29 ± 6.88	.899
	Metastatic LN, *n*	1.67 ± 1.55	1.89 ± 2.15	.595
	Ratio (%)	23.05 ± 22.23	26.15 ± 31.04	.636
Level V	Harvested LN, *n*	6.36 ± 5.21	5.63 ± 4.48	.597
	Metastatic LN, *n*	0.36 ± 0.86	0.63 ± 0.83	.258
	Ratio (%)	7.99 ± 22.19	11.98 ± 17.38	.489

All values in the table are mean ± SD

*LN* lymph node, *n* number, *SD* standard deviation

Scores for VSS and subjective sensory change in the neck area were not significantly different between the two groups (Table 4). Total cosmetic satisfaction score was significantly lower (more satisfied) in the transverse incision group than in the hockey stick group (6.35 ± 5.07 vs. 9.08 ± 4.81; *P* = 0.029) and was also significantly lower for overall satisfaction with the scar (1.54 ± 1.06 vs. 2.29 ± 0.86; *P* = 0.003) and satisfaction with the neck contour (1.43 ± 0.91 vs. 2.08 ± 0.88; *P* = 0.004). Scores for scar consciousness and sensory change were also lower in the transverse incision group than in the hockey stick group; although, this was not statistically significant.

**Table 4:** Comparison of cosmetic satisfaction and sensory change scores in patients who underwent lateral neck dissection

Parameter	Transverse Incision group *n* = 62	Hockey stick Incision group *n* = 35	*P* value
Vancouver Scar Scale (VSS) Score^a^	0.50 ± 0.93	0.45 ± 0.52	.881
Total Cosmetic Satisfaction Score^a^	6.35 ± 5.07	9.08 ± 4.81	.029*
Scar satisfaction	1.54 ± 1.06	2.29 ± 0.86	.003*
Contour satisfaction	1.43 ± 0.91	2.08 ± 0.88	.004*
Scar noticeable	0.88 ± 1.02	1.08 ± 1.01	.394
Try to conceal the scar	0.89 ± 1.04	1.16 ± 1.09	.287
Scar influences choice of clothes	0.89 ± 1.10	1.25 ± 1.18	.196
Frequency of thinking about scar	0.91 ± 0.93	1.21 ± 0.88	.182
Sensory Change Score^a^	1.71 ± 1.17	2.01 ± 1.13	.431
Survey period (months after surgery)^b^	16.4 ± 3.4	18.2 ± 2.1	.137

All values in the table are mean ± SD

*P < 0.05

^a^Lower scores mean greater satisfaction

^b^VSS, cosmetic satisfaction, and sensory change were evaluated on the same day

## Discussion

Improving quality of life has been a major goal in the treatment of differentiated thyroid carcinoma, as patients have a good prognosis [13, 14]. In general, patients are deeply concerned about a visible scar in the neck [11]. Recently, remote-access incisions, such as the transaxillary, axillo-breast, and postauricular facelift incisions, have been developed for LND of thyroid cancer, with the aim of concealing the scar in the neck area, and thereby demonstrating the interest of both surgeons and patients in cosmetic results [6, 15–17]. Even in the conventional transcervical approach, there have been attempts to reduce the scar length or to design a less noticeable skin incision for better cosmesis [9].

Various incisions are used for transcervical LND in the treatment of thyroid cancer with lateral compartment lymph node metastasis. A hockey stick incision was introduced by Lahey et al. in 1940 [18]. The hockey stick incision enables good exposure of the parotid area and neck levels I and II (Fig. 1b). However, a vertical portion of the incision lateral to the sternocleidomastoid muscle crosses the Langer’s tension line. To avoid the vertical incision, MacFee suggested parallel transverse incisions for radical neck dissection in 1951 [19]. However, this approach has the disadvantages of limited exposure and increased operative time, especially in patients with short and/or obese necks [20, 21]. L-shaped hockey stick incisions and modified MacFee incisions have traditionally been used to expose the upper neck, including levels I and II, during LND for thyroid cancer. However, smaller incisions or single transverse incisions are being utilized more frequently as a result of increasing concerns for better cosmesis. The development of energy devices, such as the ultrasonic scalpel, has enabled surgeons to dissect the upper neck relatively easily without the need for a large incision. An extended single transverse incision, which is the extension of a transverse incision for thyroidectomy, does not cross the skin-tension line, and thus, good cosmetic results are anticipated. However, there have been concerns that this approach can result in incomplete excision and worse oncological outcomes [7]. In particular, surgeons may have concerns about incomplete dissection of level II using this approach.

In our institution, we use either an L-shaped hockey stick incision or a single low transverse incision for total thyroidectomy and concomitant lateral compartment neck dissection. Because the hockey stick incision invades the resting skin-tension line, we recently started to favor the low transverse incision. In our institution, the low transverse incision is placed one finger-breadth superior to the suprasternal notch; this is more inferior to the modified extended Kocher incision, which is at the level of cricoid cartilage [7]. The advantage of the low transverse incision over the modified extended Kocher incision is that the scar can be concealed by clothes with collars.

To date, cosmetic and functional results of the low transverse incision approach have not yet been evaluated thoroughly. One preliminary study compared the cosmetic results of the modified extended Kocher incision and the apron incision, with a short follow-up period [7]. However, the study analyzed only a limited number of factors, including the presence of hypertrophic scarring and stretching, the use of silicone gel therapy, and scar revision [7]. To our knowledge, the current study is the first to evaluate the scar resulting from a single low transverse incision in the treatment of PTC with lateral neck metastasis. We analyzed the objective and subjective, cosmetic and functional results of the low transverse incision compared to the hockey stick incision using a validated objective scar measurement (the VSS); a subjective scar assessment for cosmetic satisfaction, scar consciousness, and sensory change; and surgical outcome measures including the number of harvested and metastatic lymph nodes at each neck level.

In the current study, there were no significant differences in operative time, complication rates, or oncological outcomes between the transverse incision and hockey stick incision groups. The number of lymph nodes harvested from the transverse incision group at neck levels II and V and the total lateral compartment were 9.8, 6.4, and 27.7, respectively. These values are in accordance with the number of lymph nodes harvested from the hockey stick incision group (9.6, 5.6, and 29.3, respectively) and the results from a published data (mean 8.3–10.3, 6.2–6.6, and 31.1–31.5, respectively) [22–24]. In addition, the mean number of metastatic lymph nodes in the transverse incision group at neck levels II and V and the total lateral compartment were 2.4, 0.4, and 5.7, respectively, which were similar to both the mean number of metastatic lymph nodes in the hockey stick incision group (2.3, 0.6, and 6.5) and the published data (1.2–2.7, 0.3–1.9, and 4.6–4.8) [22–24]. These results suggest that surgical accessibility to the upper neck level is comparable between the single low transverse incision and the hockey stick incision in LND (Fig. 2b).

Objectively, the mean VSS score in the transverse incision group was 0.50, with no statistical difference to the VSS score (mean 0.45) in the hockey stick incision group. These scores are lower than those reported after transcervical thyroidectomy without LND in a study by O’Connell et al. (VSS score mean 2.5) [9]. This difference might be attributable to patient factors and/or a difference in observer or surgical technique, such as suture tension.

Subjectively, patient satisfaction with scar and neck contour was significantly higher in the transverse incision group. Also, scar consciousness (measured by whether the patient thinks others notice the scar, whether the patient tries to conceal the scar, whether the scar influences the patient’s choice of clothes, and how often the patient thinks about the scar) was less in the transverse incision group; although, it did not reach statistical significance. Taken together, these results confirm the superior cosmesis of the low single transverse incision, which is considered to be the main advantage of lateral neck dissection.

There are some limitations to the current study. This study lacks randomization for the two incision approaches, and the sample size was small. Recently, we performed more cases of transverse incision than hockey stick incision, due to its cosmetic superiority. However, the mean follow-up periods are not significantly different between the two groups, and all the neck dissections in this study were performed after the period of learning curve of the surgeon (K.T.). A future randomized study including a larger number of patients is necessary to overcome these limitations. In addition, the questionnaire for cosmetic satisfaction and scar consciousness has not yet been validated. However, the scores for cosmetic satisfaction and scar consciousness correlated significantly with VSS in our previous study [11, 25]. Another limitation is that the study population included only Koreans. It is well known that ethnicity influences the formation of hypertrophic scars and keloids [8]. Therefore, the results of this study cannot be easily translated to patients in other ethnic groups and in other countries.

## Conclusions

In conclusion, a single low transverse incision for LND and total thyroidectomy provides equivalent surgical outcomes and superior cosmetic outcomes compared to those in a hockey stick incision in the treatment of PTC with lateral compartment lymph node metastasis.

## Abbreviations

LND: Lateral neck dissection
MRND: Modified radical neck dissection
N: Node
PTC: Papillary thyroid carcinoma
RAI: Radioactive iodine
T: Tumor
TG: Thyroglobulin
TNM: Tumor, node, metastasis
TSH: Thyroid-stimulating hormone
VSS: Vancouver Scar Scale
